# Post-traumatic Stress Disorder: The Influence of the Environmental Context and Analysis of Oxidative Stress and Inflammatory and Glycemic Markers in Women Living in Kurdistan Regional Government-Iraq

**DOI:** 10.7759/cureus.56661

**Published:** 2024-03-21

**Authors:** Husni M Hasan, Suad Y Alkass, Daniele S Persike

**Affiliations:** 1 Department of Medicinal Chemistry, Department of Chemistry, College of Pharmacy, College of Science, University of Duhok, Duhok, IRQ; 2 Department of Medicinal Chemistry, College of Pharmacy, University of Duhok, Duhok, IRQ

**Keywords:** genocide, yazidi, biochemical parameters, inflammation, antioxidants, oxidative stress, environmental context, post-traumatic stress disorder

## Abstract

Background

Internally displaced persons (IDP) camps are still home to a large number of female survivors of the Yazidi genocide carried out in Iraq in 2014 by the Islamic organization known as the Islamic State of Iraq and Syria (ISIS). Many of these women suffer from a persistent form of post-traumatic stress disorder (PTSD), which can last for many years. On the other hand, little is known about the intricate etiology of PTSD.

Objectives

In this observational cross-sectional study, the biochemical parameters, including inflammatory and oxidative stress (OXS) markers, were evaluated in two groups: the case group (women with newly diagnosed PTSD) and the control group (apparently healthy women). Furthermore, how the environment impacts the biochemical and OXS parameters of people not diagnosed with PTSD but living in IDP camps was also analyzed.

Materials and methods

The PTSD group (n=55, age=30.0 years) was made up of women survivors of genocide-related events living in IDP camps in the Kurdistan region of Iraq. The studied parameters in the PTSD group have been compared to two healthy control groups: (1) internal control group (n=55, age=28.1 years): healthy women living inside the IDP camps; and (2) external control group (n=55, age=28.3 years): healthy women living outside the IDP camps. The diagnosis of PTSD was conducted using a validated Kurdish version of the PTSD Diagnostic and Statistical Manual of Mental Disorders, Fifth Edition (DSM-5) (PCL-5) scale. Blood samples were collected to determine the level of glycated hemoglobin (HbA1c) and the concentrations of fasting serum glucose (FSG), C-reactive protein (CRP), ceruloplasmin (CP), 8-hydroxydeoxyguanosine (8-OHdG), glutathione (GSH), malondialdehyde (MDA), protein carbonyls (PC), and catalase (CAT) activity.

Results

Women with PTSD presented increased values of FSG (4.41%, p<0.05), HbA1c (4.74%, p<0.05), and CRP (114.29%, p<0.05), as well as increased levels of 8-OHdG (185.97%, p<0.001), CP (27.08%, p<0.001), MDA (141.97%, p<0.001), and PC (63.01%, p<0.001), besides increased CAT activity (121.5%, p<0.001), when compared with the control groups. A significant reduction of GSH (-20.33%, p<0.05) was observed in PTSD patients as compared to the external control group. In relation to the internal control group, women diagnosed with PTSD presented significantly increased levels of FSG (3.88%, p<0.05), HbA1c (2.83%, p<0.05), CRP (77.97%, p<0.05), and PC (41.3%, p<0.05), as well as increased levels of 8-OHdG (118.84%, p<0.001), CP (22.72%, p<0.001), MDA (90.67%, p<0.001), and CAT activity (55.31%, p<0.001). Healthy individuals residing in IDP camps, compared with external healthy control, presented significantly elevated levels of 8-OHdG (30.68%, p<0.001), MDA (26.91%, p<0.001), PC (15.37%, p<0.001), and CAT activity (42.62%, p<0.001).

Conclusion

Our findings indicate that PTSD significantly influences glycemic, inflammatory, oxidant, and antioxidant parameters, as evidenced by increased levels of FSG, HbA1C, CRP, PC, MDA, 8-OHdG, and CP, as well as increased CAT activity and a reduced GSH concentration in the PTSD group in comparison to the external control group. Additionally, our results suggest that the environmental context in IDP camps by itself can potentially affect oxidant and antioxidant parameters, as evidenced by the increased concentrations of 8-OHdG, MDA, and PC and increased CAT activity found in individuals not diagnosed with PTSD but living inside the camps.

## Introduction

Post-traumatic stress disorder (PTSD) is an extreme anxiety-related health problem involving physical and psychological signs and symptoms caused by exposure to severe trauma, which can have profound negative consequences for individuals [1,2]. The development and persistence of PTSD can be attributed to several environmental and biological risk factors [3]. It was estimated that about six out of every 100 people will experience PTSD at some point in their lives [4].

The situation of the Yazidi community became particularly dramatic after the Islamic State of Iraq and Syria (ISIS) attacked Sinjar, the homeland of Yazidi forefathers in northwestern Iraq, in 2014. As a result, thousands of Yazidis took refuge in internally displaced persons (IDP) camps and villages in Duhok City, Iraq. PTSD has a higher prevalence among women veterans of war and girls compared to women civilians and men veterans [5]. Hence, female individuals in IDP camps are at greater risk of developing PTSD [6]. In the displaced families, most women and girls have been affected by the genocide-related events after the ISIS attack in Iraq. For the Yazidis, the ISIS invasion was the 74th genocide in succession across 800 years. This perpetration is classified as a crime against humanity and as a war crime [7,8].

In today's medical field, the criteria for diagnosing PTSD focus primarily on clinical symptoms indicative of the disease, such as behavioral, cognitive, and emotional aspects [9]. There is a lack of biomarkers associated with a variety of anxiety-related health conditions, including PTSD.

Hence, it is essential to identify biochemical markers for individuals with health problems whose physical and psychological signs and symptoms are related to PTSD, as the disease shares several signs and symptoms with other psychiatric diseases. Typically, a biochemical marker could be applied to guide the clinical diagnosis of PTSD [10].

Association of PTSD with oxidative stress (OXS)

Numerous studies have clearly observed that the production of reactive oxygen species (ROS) is significantly involved in the development of various neuropsychiatric conditions, suggesting that it might play a role in the pathophysiology of PTSD [11-13]. Data coming from cross-sectional studies have identified significant changes in blood antioxidant enzyme activity and OXS-related gene expression between individuals with and without PTSD [12].

In the human body, under typical physiological life situations, there is a dynamic equilibrium between the generation of ROS and their removal through the collective effort of antioxidants to protect against the damaging consequences of OXS achieved through activating antioxidant enzymes [14,15].

Free radicals have limited lifetimes and low quantitative levels, making it difficult to quantify them directly. OXS assessment is performed by measuring reactive metabolites of oxygen and nitrogen species (ROS/RNS), antioxidant levels, and antioxidant enzyme activities [16]. In our study, oxidative biomarkers linked to oxidative damage, including lipid peroxidation, protein, and DNA oxidation products, were quantified. The assessment of malondialdehyde (MDA) levels is a specialized method to evaluate lipid peroxidation. Meanwhile, assessing protein and DNA oxidation involves quantifying the amount of protein carbonyls (PC) and 8-hydroxydeoxyguanosine (8-OHdG), respectively [17].

Assessment of the levels of antioxidant enzymes such as ceruloplasmin (CP), glutathione (GSH), and catalase (CAT) activity is of crucial importance to know whether alterations in those antioxidant enzymes are directly associated with PTSD disease and its development.

The present study aimed to determine the concentration of biochemical parameters and OXS status in women survivors of genocide-related events living in IDP camps in Duhok, Kurdistan Regional Government-Iraq (KRG-Iraq).

## Materials and methods

Ethical considerations

The study was approved by the Scientific Committee of the College of Science, University of Duhok, and the Medical Ethical Committee of the Directorate of Health in Duhok Governorate (reference number 22062021-6-3).

Study design

This observational cross-sectional study was carried out between November 2021 and June 2022 at the IDP camps in Duhok Province, KRG-Iraq. The newly diagnosed PTSD group involved Yazidi women who have endured persecution and violence at the hands of ISIS and are living with their families in Kabartu, Khanke, Sharia, Bajet Kandala, and Qadia IDP camps after being displaced from their ancestral homes in Sinjar. The internal control group involved apparently healthy women living in the same camps. The external healthy control group involved apparently healthy women selected from the college staff and their relatives in Duhok City who were not living inside the camps.

Sampling technique

Each participant enrolled in this study provided formal informed consent after the importance and purpose of the study were clearly explained. A pre-validated questionnaire was designed by the authors to meet the study's specific needs, and information on age, alcohol intake, and smoking habits, besides prior medical history (including cardiac, hepatic, renal, and any other disorders), was collected from all the respondents who fulfilled the inclusion criteria.

The exclusion criteria were pregnancy; the presence of chronic diseases, including diabetes mellitus, cancer, and liver, heart, and renal diseases; endocrine disorders; and a family history of neuropsychiatric disorders prior to the genocide, smokers, alcohol consumers, and people who were taking supplements.

Study groups

A group of 130 women who had not been diagnosed with PTSD was interviewed. After applying the exclusion criteria, 110 apparently healthy women remained in the study. The healthy women (n=110) were classified into the internal control group (n=55, mean age=28.1 years) and external control group (n=55, mean age=28.3 years), based on whether they were living inside or outside the IDP camps. The participants in the external control group were selected from college staff, college staff relatives, and friends. A total of 62 women were diagnosed with PTSD. After applying the exclusion criteria, 55 women with PTSD remained enrolled in the PTSD group (n=55, mean age=30.0 years). Hence, the participants were classified into three groups as follows: (1) external control group: apparently healthy women living outside the IDP camps; (2) internal control group: apparently healthy women living inside the IDP camps; and (3) PTSD group: women diagnosed with PTSD living inside the IDP camps.

Assessment instruments

The diagnosis of PTSD was done by a psychiatrist according to the validated Kurdish version of the PTSD diagnostic criteria of the Diagnostic and Statistical Manual of Mental Disorders, Fifth Edition (DSM-5) (PCL-5) checklist [18]. PCL-5 is a 20-item self-assessment questionnaire that measures PTSD symptoms as listed in the DSM-5 according to a 0-4 rating scale; for each item, a score of 2 or above is regarded as clinically relevant. A PCL-5 cut-point of 33 is recommended to consider PTSD. The participants were chosen by a random sampling method from camps of IDP located in the Duhok Governorate within the KRG-Iraq. Individuals with PTSD were newly diagnosed and were not receiving any psychiatric medication.

Collection of blood samples

Venous blood samples (10 mL) were collected after an overnight fast (12-14 hours) from the 55 PTSD patients and 110 healthy women. Immediately after collecting the blood sample, 2 mL of whole blood was placed in a lavender-top tube containing EDTA to determine the HbA1c level. The remaining blood sample (8 mL) was placed in a gel-containing tube and allowed to clot for 10-20 minutes at room temperature and then centrifuged at 1000 g for 20 minutes. The serum was separated and used to analyze all the other required parameters. The assessment of the biochemical parameters was conducted in the Department of Chemistry, College of Science, University of Duhok, Kurdistan region of Iraq.

FSG, CRP, and CP measurements were performed on the same day of the sample collection. The remaining serum was distributed into Eppendorf tubes and kept frozen at -28°C until the assessment of oxidative stress parameters.

Biochemical analysis

The HbA1c level (%) was performed by a high-performance liquid chromatography (HPLC) assay using whole blood. The ADAMS™ A1C HA-8180v Analyzer (ARKRAY Co. Ltd., Inc., US) was used.

The hexokinase method was used to investigate the concentrations of FSG using a commercially available kit catalog number (04404483190) from Roche/Hitachi Diagnostics GmbH/Germany Cobas® 6000. Turbidimetry was applied to determine the serum concentration of CRP and CP using commercial measurement kits supplied by Roche Diagnostics GmbH/Germany, Cobas® 6000 (Roche and Hitachi High-Technologies Corporation, Tokyo, Japan) under catalog numbers (07876033190) and (08105553190), respectively.

The CAT activity and serum concentration of PC and MDA were measured using commercial human sandwich enzyme-linked immunosorbent assay (ELISA) kits (BT LAB Test, Bioassay Technology Laboratory, China) under catalog numbers (E3053Hu), (E1426Hu), and (E1371Hu), respectively. The assay has a minimum detectability of 1.12 KU/L for CAT, 1.07 ng/mL for PC, and 0.14 nmol/mL for MDA, which are below the range found in healthy human controls. The test's measurements' intra-assay and inter-assay coefficients of variation (CVs) were <8% and <10%, respectively.

The serum concentrations of 8-OHdG and GSH were measured using commercial ELISA kits (Elabscience Biotechnology Inc., USA) under catalog numbers (E-EL-0028) and (E-EL-0026), respectively.

Statistical analysis

All data were analyzed using the Statistical Package for the Social Sciences (SPSS, version 26.0; IBM SPSS Statistics for Windows, Armonk, NY) program software. The results were presented as the mean ± standard error (SE). Skewness and kurtosis tests were performed on all the study groups to assess their normal distribution. A significance level of p<0.05 was considered statistically significant. The mean values between groups were compared using a one-way ANOVA. In contrast, the percentage of changes between the PTSD and control groups were compared using an independent t-test.

## Results

Effect of PTSD on the studied biochemical and hematological parameters

The mean values shown in Table 1 indicate that most of the studied parameters were significantly increased in the PTSD group compared to the external and internal control groups, except serum GSH, which was not significantly decreased.

**Table 1:** Mean values of the biochemical and oxidative stress parameters in the studied groups. The comparison of fasting serum glucose (FSG), glycated hemoglobin (HbA1c), catalase activity (CAT), ceruloplasmin (CP), C-reactive protein (CRP), glutathione (GSH), 8-hydroxydeoxyguanosine (8-OHdG), malondialdehyde (MDA), and protein carbonyls (PC) within the studied groups was conducted using a one-way ANOVA. Results with p>0.05 were regarded as non-significant, while significant and highly significant findings were marked with * for p<0.05 and ** for p<0.001, respectively.

Variables	Studied groups (Mean ± SE)			p value
	External control (n = 55)	Internal control (n = 55)	PTSD (n = 55)	
FSG (mg/dL)	89.10 ± 0.73	89.73 ± 0.8	93.21 ± 1.05	0.004*
HbA1c (%)	4.85 ± 0.04	4.94 ± 0.03	5.08 ± 0.06	0.002*
CAT (KU/L)	148.69 ± 3.61	212.06 ± 6.61	329.35 ± 16.32	<0.001**
CP (mg/dL)	18.87 ± 0.31	19.54 ± 0.53	23.98 ± 0.73	<0.001**
CRP (mg/dL)	0.98 ± 0.03	1.18 ± 0.06	2.10 ± 0.13	<0.001**
GSH (µg/mL)	11.46 ± 0.94	10.06 ± 0.58	9.13 ± 0.54	0.067
8-OHdG (ng/mL)	8.41 ± 0.33	10.99 ± 0.35	24.05 ± 1.72	<0.001**
MDA (nmol/mL)	19.25 ± 0.49	24.43 ± 1.23	46.58 ± 1.76	<0.001**
PC (ng/mL)	133.35 ± 4.47	153.84 ± 4.38	217.37 ± 12.44	<0.001**

Effect of PTSD on serum glucose and glycated hemoglobin

**Figure 1:**
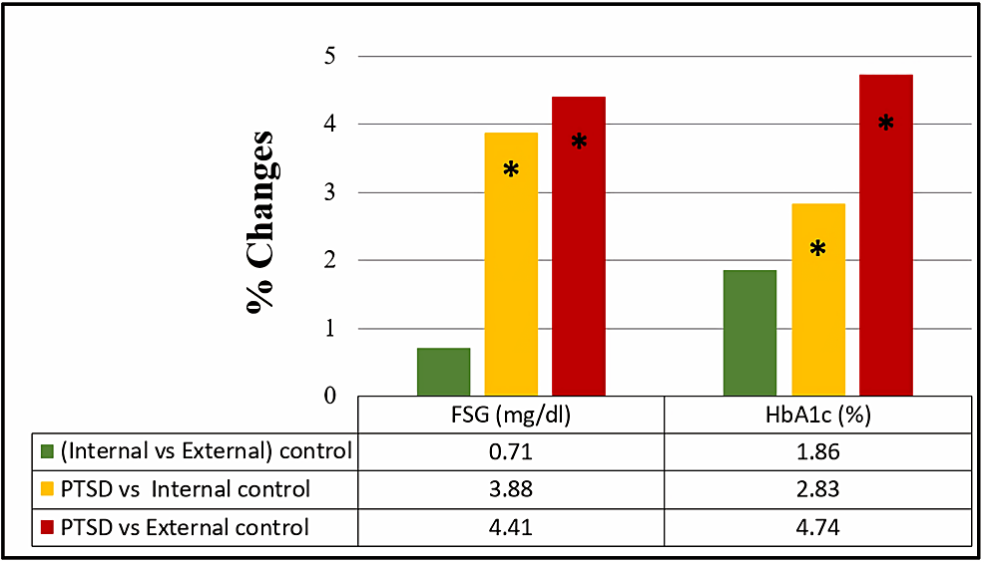
Percentage of change in fasting serum glucose and glycated hemoglobin among the studied groups. The comparison of fasting serum glucose (FSG) and glycated hemoglobin (HbA1c) within the studied groups was assessed through an independent t-test. Results with p>0.05 were regarded as non-significant, while significant and highly significant findings were marked with * for p<0.05 and ** for p<0.001, respectively.

A comparison of mean values among the studied groups indicates a significant increase (p<0.05) in FSG and HbA1c when the PTSD group was compared with both external and internal control groups, as illustrated in Figure 1.

FSG and HbA1c exhibited a significant increase of 3.88% and 2.83%, respectively, in the PTSD group compared to the internal control group. Moreover, further significant increases of 4.41% and 4.74% in FSG and HbA1c, respectively, were noticed in comparing the PTSD group with the external control group.

Effect of PTSD on CRP

**Figure 2:**
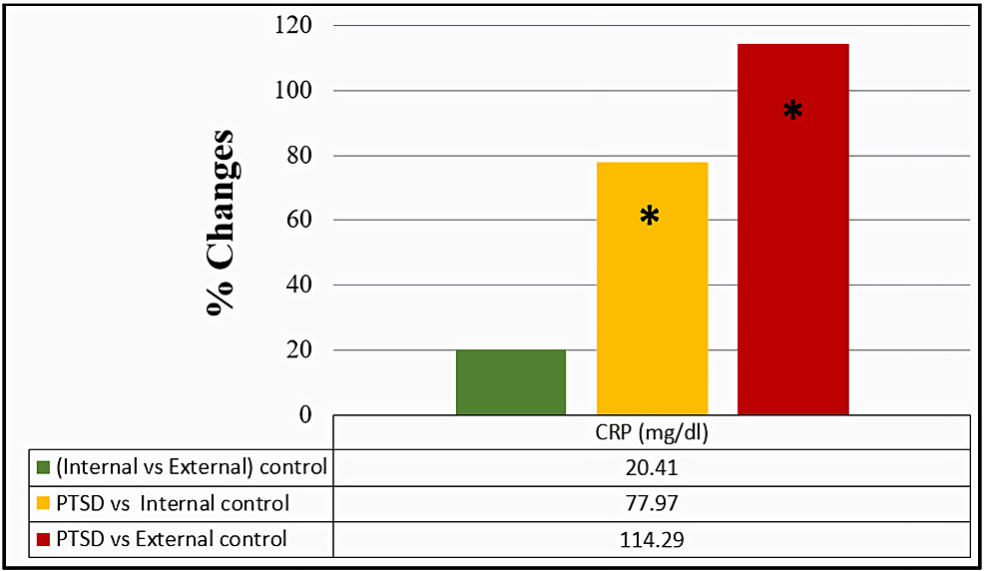
Percentage of change in serum C-reactive protein among the studied groups. The comparison of C-reactive protein (CRP) within the studied groups was assessed through an independent t-test. Results with p>0.05 were regarded as non-significant, while significant and highly significant findings were marked with * for p<0.05 and ** for p<0.001, respectively.

The concentration of CRP in the PTSD group exhibited a significant increase of 77.97% compared to the internal control group, further increasing by 114.29% when the PTSD group was compared with the external control group, as illustrated in Figure 2.

Effect of PTSD on the by-products of oxidation

**Figure 3:**
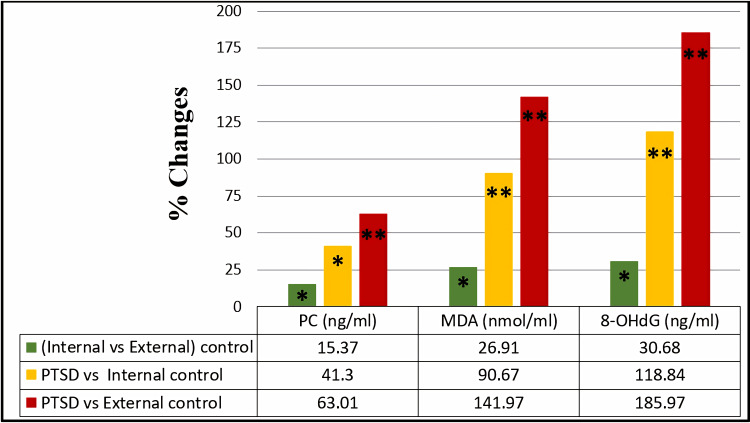
Percentage of change in serum protein carbonyl, serum malondialdehyde, and 8-hydroxydeoxyguanosine among the studied groups. The comparison of serum protein carbonyl (PC), serum malondialdehyde (MDA), and 8-hydroxydeoxyguanosine (8-OHdG) within the studied groups was assessed through an independent t-test. Results with p>0.05 were regarded as non-significant, while significant and highly significant findings were marked with * for p<0.05 and ** for p<0.001, respectively.

Regarding the oxidative stress parameters, the results showed a significant increase in PC, MDA, and 8-OHdG levels in the PTSD group by 41.3%, 90.67%, and 118.84%, respectively, as compared with the internal control group. Further significant increases of 63.01%, 141.97%, and 185.97% were observed for those same parameters in the PTSD group, compared with the external control group, as shown in Figure 3.

A significant increase of PC, MDA, and 8-OHdG by 15.37%, 26.91%, and 30.68%, respectively, was observed in the internal control group compared to the external control group.

Effect of PTSD on antioxidant parameters

**Figure 4:**
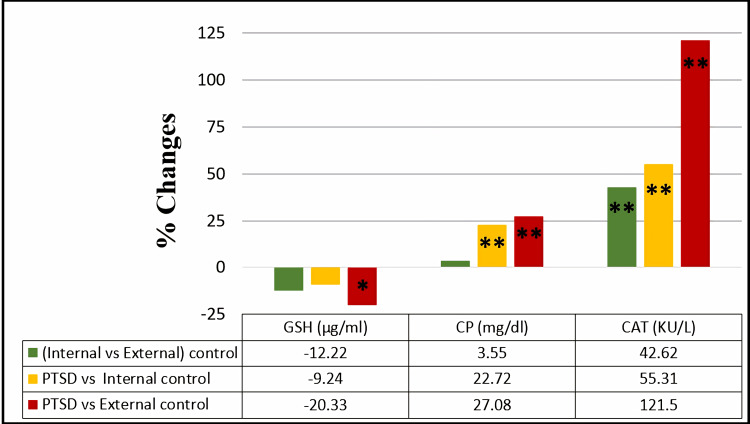
Percentage of change in serum glutathione, serum ceruloplasmin, and serum catalase activity among the studied groups. The comparison of glutathione (GSH), ceruloplasmin (CP), and catalase activity (CAT) within the studied groups was assessed through an independent t-test. Results with p>0.05 were regarded as non-significant, while significant and highly significant findings were marked with * for p<0.05 and ** for p<0.001, respectively.

Concerning the percentage of changes in the mean values of antioxidant parameters, there was a highly significant increase in CP and CAT activity in the PTSD group by 22.72% and 55.31%, respectively, compared to the internal control group. Additionally, further significant increases in CP and CAT activity by 27.08% and 121.5%, respectively, and a significant decline in GSH level by -20.33% were observed in the PTSD group compared to the external control group, as shown in Figure 4.

Additionally, there was a highly significant increase in CAT activity by 42.62% within the internal control group compared to the external control group.

## Discussion

The genocide perpetrated by ISIS in Sinjar, northern Iraq, has resulted in numerous deaths and long-lasting mental health issues, including PTSD, particularly among Yazidi women and girls. The aforementioned effect is observed in surviving female individuals [19]. Since the ISIS attack occurred in 2014 and many Yazidis are still living inside IDP camps without knowing when they will be able to return to their homeland, some studies have started to investigate the physical and mental health status of the Yazidi women who have survived the genocide-related events [20].

The present cross-sectional study is the first study to analyze the impact of PTSD on biochemical and OXS parameters among Yazidi women survivors of genocide-related events after the ISIS attack on Iraq in 2014. Furthermore, the study presents results showing for the first time that the environment may impact the OXS and biochemical parameters of people not diagnosed with PTSD living in an IDP camp along with people diagnosed with PTSD.

Effect of PTSD on serum glucose and glycated hemoglobin

PTSD could be considered a risk factor for developing type 2 diabetes and insulin resistance via several conceivable mechanisms [21]. PTSD is associated with elevated levels of inflammatory markers, including CRP, interleukin-6 (IL-6), and tumor necrosis factor-alpha (TNF-α), that could exacerbate insulin resistance [22,23]. In addition, PTSD is linked to changes in the hypothalamic-pituitary-adrenal axis and cortisol level [24], which have been demonstrated to be associated with visceral obesity and insulin resistance [25]. Furthermore, PTSD is connected to a higher body mass index (BMI), disrupted sleep patterns, and lifestyle modifications, such as prolonged inactivity and unhealthy dietary choices, all of which collectively contribute to the onset of diabetes [26].

Similar results were obtained in the present study since FSG and HbA1c were significantly increased when the PTSD group was compared with both control groups. The percentage of FSG and HbA1c changes in PTSD patients compared with the external control group was higher than when compared with the internal control group. According to that, we can consider that higher levels of FSG and HbA1c in healthy women residing in IDP camps might be associated with changes in diet and physical activity, including sedentary behaviors. The results obtained are indicative of impaired glucose metabolism, which may eventually contribute to the development of pre-diabetes and type 2 diabetes [27]. Additionally, the phenomenon of displacement, homelessness, conflict, and residing in precarious circumstances in IDP camps can affect mental health and potentially result in heightened levels of stress. Stress, in turn, induces the secretion of hormones such as cortisol and adrenaline, influencing the functioning of insulin in the body and potentially elevating glucose levels in the bloodstream [28].

Effect of PTSD on CRP

A marker of inflammation in the body, CRP, has been linked to mental health problems, such as PTSD [29]. Several studies indicate that going through trauma could cause the immune system to become more active, including producing an inflammatory response [30,31].

The current study expands on these previous findings by demonstrating a significantly elevated CRP level in female Yezidi community survivors with PTSD. When the mean values of the differences in CRP concentrations in survivors with PTSD and healthy control groups were compared, a significant increase in CRP levels in individuals with PTSD was found. The percentage of CRP change in PTSD patients compared with the external control group was higher than when compared with that of the internal control group. According to these results, we can hypothesize that a higher level of CRP in the internal control group (apparently healthy women) could be an indicator that people living on IDP campuses might be indirectly affected by PTSD. The women included in the internal control group were not diagnosed with PTSD; however, they shared the same environment as those diagnosed with PTSD. The hypothesis mentioned is supported by previous research showing associations between contextual factors and CRP levels in developing mental health problems in refugees [32].

Effect of PTSD on the production of OXS parameters

In this study, higher levels of oxidation products, including serum MDA, PC, and 8-OHdG, were observed in the PTSD group compared with the control groups. The by-product of lipid peroxidation (MDA) was found to be twofold elevated in the PTSD group compared to the internal control group. The current result is in line with other studies indicating that civilian earthquake survivors and the military population developed PTSD, which was associated with an increase in the level of MDA biomarkers of lipid peroxidation [12,33]. Furthermore, two animal studies have shown an association between PTSD and lipid peroxidation in rats with PTSD, showing elevated levels of MDA in various organ tissues when compared to healthy rats [34,35].

Besides the lipid oxidation processes, reactive species often cause proteins to be oxidized, resulting in the production of carbonyl groups on amino acid side chains, which can be used as biomarkers in human disease detection [36]. In this study, we found that oxidative damage to proteins occurred, as evidenced by an elevation of PC levels in women with PTSD compared with the control groups. This result demonstrates that increasing serum PC levels are associated with PTSD. A different result was found by Čeprnja et al. [37], showing that PC levels were reduced in male soldiers with PTSD compared with healthy controls. Another study, done by Koike et al., showed that there is a link between mental health problems and protein oxidation, as they found that PC levels were increased in patients with schizophrenia compared to healthy individuals [38]. An animal model study in rats showed increased PC concentrations in myocardial tissue, the liver, and the cerebral cortex in rats with PTSD compared to healthy rats without PTSD [34].

In order to investigate the effects of PTSD on OXS, our study has analyzed the levels of 8-OHdG, a marker of DNA oxidative damage. This study showed that 8-OHdG was markedly elevated in women with PTSD when compared with apparently healthy women. Our main findings indicated that an increase in serum 8-OHdG concentration was associated with PTSD. The present findings are consistent with previous studies showing that increased 8-OHdG serum concentrations [39] and increased 8-OHdG concentrations using DNA extracted from the whole blood were found [40] in the participants with PTSD. In addition, a meta-analysis study demonstrated that the concentration of 8-OHdG was significantly increased in individuals diagnosed with mental health problems, including schizophrenia and bipolar disorder [41].

Additionally, to our knowledge, this is the first study to show a significant increase in MDA, PC, and 8-OHdG concentrations in healthy individuals residing in IDP camps compared with healthy individuals not living in IDP camps. This finding indicated that differences in exposures over the life course among individuals residing in IDP camps, coupled with the impact of homelessness and the refugee process, result in increased oxidative stress, leading to oxidative damage of lipids, proteins, and DNA, which can potentially modify biological pathways to disease. It could result in distinct disease profiles among IDP individuals. As we hypothesized from this outcome, IDP individuals are at higher risk of suffering and developing mental health problems, including PTSD.

Effect of PTSD on antioxidant parameters

The current study demonstrated that there was a depletion in the non-enzymatic antioxidant GSH concentration in PTSD women when compared to the external healthy control group. These findings are similar to those of Borovac et al. and Petrovic et al., who showed GSH depletion in humans with PTSD [42] and in animal rats with PTSD [35]. Our findings suggest that, when OXS persists for an extended period and the body's cellular defense mechanisms cannot effectively combat these processes, the available free GSH decreases, resulting in irreversible cellular degeneration and cell mortality [43]. Depletion in GSH levels has been noted in individuals with various psychiatric conditions, including schizophrenia [44,45] and bipolar disorder [45].

In contrast to decreased GSH, there was an elevation in the concentration of non-enzymatic antioxidant CP in PTSD individuals when compared to healthy control groups. Ceruloplasmin has an essential role as a protective agent against oxidative stress and in the control of lipid oxidation [46]. Therefore, serum CP levels in PTSD individuals are elevated to overcome the elevation in MDA levels in order to protect the body from lipid peroxidation. Elevated levels of CP in the bloodstream have been observed in individuals with other psychiatric disorders [47,48].

Besides the CP, an elevation in serum CAT activity among PTSD women compared to healthy control groups was also observed. Catalase plays a crucial role as an antioxidant enzyme, which contributes significantly to maintaining cellular redox balance by facilitating the breakdown of hydrogen peroxide in the body [49]. We suggest that the consequent high CAT activity in PTSD patients might be a response to and overcome the increased production of ROS due to OXS in PTSD. Our data corroborate with previous studies conducted on psychiatric illness, including major depressive disorder and schizophrenia, showing high activity of CAT in individuals with major depressive disorder [50] and schizophrenia [51].

**Figure 5:**
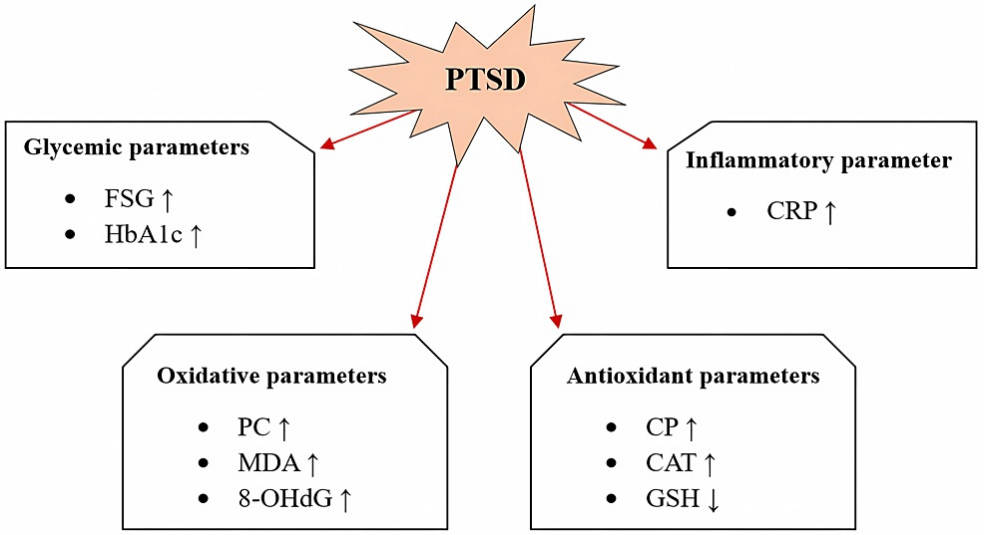
Impact of post-traumatic stress disorder on glycemic, inflammatory, oxidative, and antioxidant parameters. The impact of post-traumatic stress disorder (PTSD) on fasting serum glucose (FSG), glycated hemoglobin (HbA1c), C-reactive protein (CRP), protein carbonyls (PC), malondialdehyde (MDA), 8-hydroxydeoxyguanosine (8-OHdG), ceruloplasmin (CP), catalase activity (CAT), and glutathione (GSH) is represented by arrows, where ↑ elevated and↓ reduced. The PTSD group was compared with the external control group. Image created by author Husni M. Hasan

The impact of PTSD on the glycemic, inflammatory, oxidative, and antioxidant parameters can be summarized, as shown in the diagram presented in Figure 5.

**Figure 6:**
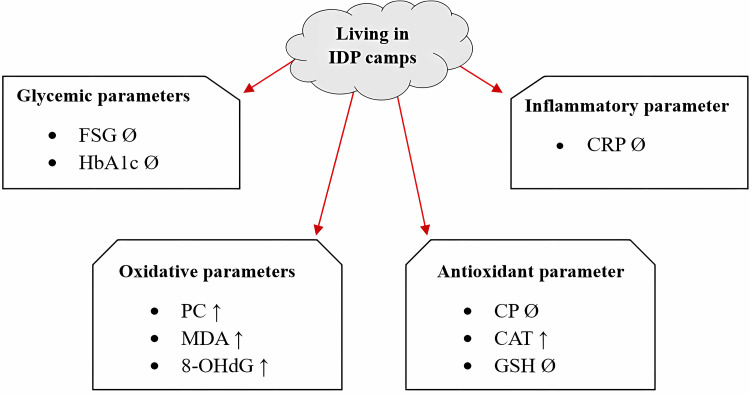
Environmental influence of living in internally displaced persons camps on glycemic, inflammatory, oxidative, and antioxidant parameters. The influence of living in internally displaced persons (IDP) camps on fasting serum glucose (FSG), glycated hemoglobin (HbA1c), C-reactive protein (CRP), protein carbonyls (PC), malondialdehyde (MDA), 8-hydroxydeoxyguanosine (8-OHdG), ceruloplasmin (CP), catalase activity (CAT), and glutathione (GSH) is represented by arrows, where: ↑ elevated, ↓ reduced, and Ø no change. The internal control group was compared with the external control group. Image created by author Husni M. Hasan

The increase in CAT activity found in the internal healthy control group was due to a response to and overcoming high ROS due to oxidative stress [52], as we found high levels of MDA, PC, and 8-OHdG in healthy IDP women. To clarify the overall effect of living in IDP camps on glycemic, inflammatory, oxidative, and antioxidant parameters compared to the external control groups, it can be summarized in a diagram presented in Figure 6.

Taken together, our results indicate that the environment inside the IDP camps might negatively affect the health status of people not diagnosed with PTSD but living inside the camps. Additionally, it has been a matter of discussion how the offspring of people suffering from PTSD could be indirectly affected by the disease, even if they have not experienced the events that have triggered PTSD in their parents. Studies done on survivors of the Holocaust and their offspring have shown that the offspring can be dramatically affected by sharing the environment with people diagnosed with PTSD [8,53]. According to those studies, parent-child characteristics and their interaction were found to contribute to the development of psychological symptoms, besides biological and even epigenetic variations. The mental health of the children appears to be influenced by the mental health issues of the parents, as well as perceived parenting, parental gender, and attachment quality [53]. Moreover, compared to having one survivor parent, having two survivor parents was associated with a higher rate of mental health issues [8]. The effect on the offspring might be present in our results, as our study was conducted 10 years after the ISIS attack in Sinjar. In our study, women survivors of the genocide and survivors of genocide-related events, such as living in displacement, were evaluated.

Medical efforts toward the prevention of mechanisms leading to oxidative stress, inflammation, and diabetes are important, according to the results presented here. Additionally, the impact of a healthy diet, the use of multivitamins including antioxidants, and the regular practice of physical exercise have not been evaluated among the Yazidi women diagnosed with PTSD.

We highlight that the 74th Yazidi genocide was perpetrated in 2014, and the offspring of the women survivors are at a higher risk of developing PTSD, besides other mental health problems. The search for biomarkers for PTSD is a major challenge, as it is a complex disease involving several physiological and biochemical mechanisms. Even animal models for studying PTSD are limited due to the complex etiology and manifestation of the disease. Perhaps the use of strategies such as omics (proteomics and genomics), promising strategies for characterizing the biological signatures of disorders that have been facilitated by the emergence of high-throughput technologies, could be beneficial in the elucidation of PTSD pathophysiology.

Study limitations

This study is not devoid of limitations. Firstly, we cannot attribute causation because of the cross-sectional design of the study. Secondly, the foundation of this research is the in-person use of professionally administered, verified questionnaires to capture the interviewee's answers. Interviewers may experience secondary trauma if they conduct interviews with groups that have experienced extreme trauma. In addition, it is not unusual to observe the discomfort an interviewee experiences when revisiting traumatic recollections. Because of this, interviewers have to have a background in psychology and must be well-trained in order to gather precise data on delicate subjects without endangering the interviewees. To conduct the interviews, a trustworthy atmosphere has to be established. In this way, only female Kurdish native interviewers were recruited. Thirdly, although those with a history of psychiatric disorders prior to the ISIS attack were excluded from the study, we lack adequate information on the trauma experiences of the participants prior to the ISIS attack. It might be difficult to determine a link between previous trauma experiences and current PTSD symptoms. Lastly, the interviews were conducted 10 years after the ISIS attack, and previous possible psychotherapy was not assessed, which might be helpful in knowing if the recovery may play a role in the investigated associations.

## Conclusions

This study showed that there is a significant effect of PTSD on oxidative stress, glycemic, and inflammatory parameters among Yazidi women survivors of the ISIS attack in Sinjar in 2014. PTSD induces oxidative damage to carbohydrates, lipids, and proteins, as proven by an elevation of 8-OHdG, MDA, and PC levels. Moreover, this study found that the antioxidants, including CP and CAT activity, were increased, while the GSH concentration was decreased in people diagnosed with PTSD in the IDP camps. PTSD patients are at higher risk for pre-diabetes, type 2 diabetes, and other comorbidities due to elevated FBS, HbA1C, and CRP.

This study found that living in IDP camps might impact people not diagnosed with PTSD by inducing oxidative damage to key biomacromolecules. Altogether, our data indicate that the people living inside the IDP camps are at greater risk of developing mental disorders and several comorbidities. It is also noticeable that people sharing the same environment as PTSD patients are urgently in need of attention regarding their mental health.
